# Does gluten free diet have more implications than treatment of celiac disease? 

**Published:** 2015

**Authors:** Ramin Talaie

**Affiliations:** *Modarress Hospital, Shahid Beheshti University of Medical Sciences, Tehran, Iran*

**Keywords:** Gluten free diet, Celiac disease, Lymphocytic deudonitis, Gluten sensitivity

## Abstract

**Aim:**

This study was aimed to evaluate symptomatic as well as histopathologic response to GFD in patients with gluten-sensitive enteropathies including celiac disease, lymphocytic duodenosis and non-specific duodenitis.

**Background:**

Gluten-free diet (GFD) is the main treatment of celiac disease. However, its impact on other disorders of gluten sensitivity spectrum is less clear.

**Patients and methods:**

In a prospective observational study in Modarres hospital Tehran, Iran, 35 patients with chronic manifestations including low BMI, diarrhea, greasy stool and bloating were evaluated using serology for anti-tTG, endoscopy and histopathology. Patients were categorized in three diagnostic groups accordingly including celiac disease (CD), lymphocytic doudenosis (LD) and non-specific duodenitis (NSD). All patients were put on a GFD for 6 months, and subjective symptomatic response, serology, endoscopy and histopathologic tests were repeated and compared with baselines and among groups.

**Results:**

Of the total 35 patients, 5 had CD (14.3%), 9 had LD (25.7%) and 21 (60%) had NSD. Bloating was the most common symptom followed by diarrhea. Majority of patients (80%) had low BMI. All symptoms alleviated following a GFD but bloating was the only significant one. A significant increase was found in total mean BMI (17.3±0.7 v.s. 17.9±0.9). Histopathologic examination showed a complete resolution in 48.5% (n=17) patients, 10 in NSD group, 4 in LD group and 3 in CD group. Final prevalence of gluten-sensitive enteropathy (LD and NSD cases that responded to GFD) was 46.6%.

**Conclusion:**

GFD may have more implications other than celiac disease. Other gluten-sensitive enteropathies, like LD and NSD, might also respond to this treatment particularly in patients with low BMI.

## Introduction

 Celiac disease is an immune-mediated enteropathy caused by a chronic inflammatory response in small intestine to consumption of gluten in genetically susceptible patients. Clinical presentations of the disease are widely various ranging from minor symptoms mimicking IBS or dyspepsia to classic symptoms of malabsorption. Classic form of the disease is diagnosed using serologic and histopathologic tests. Anti-tissue transglutaminase antibody (anti-tTG) as the most sensitive and specific serology study is used in suspected cases (1-4). A positive serology study must be followed by endoscopy of small intestine. Villous atrophy, crypt hyperplasia and increased intraepithelial lymphocytes (IELs, the threshold is > 25 lymphocytes in 100 entrocytes) are main histopathologic findings for confirmation of CD. Genetic studies can also confirm the diagnosis of CD with detecting specific human leukocyte antigen (HLA) class II genes (5, 6). 

 While recent studies have shown a marked rise in the incidence of CD with an estimated prevalence of 1% in general population, much attention has been drawn to some borderline cases in which gluten seems to play a role in causing the symptom while they don’t fulfill the serology and histologic criteria of classic CD. This heterogeneous group commonly referred as “non-celiac gluten sensitivity” generally show symptoms milder than classic CD and they seem to respond at least symptomatically to a gluten-free diet and their histopathologic study shows milder changes in villus architecture (7-11). Lymphocytic doudenosis is a subgroup of NSGS defined by increased IELs in the small intestine biopsy in the absence of other histopathologic features of the CD and negative serology (6). Although LD has more etiologies than gluten sensitivity, studies have shown that a considerable number of cases with this condition are included in the NSGS group (12). 

 Excluding gluten from diet is the only treatment known for CD so far and is indicated in all cases of confirmed CD with proved beneficial effects on symptoms, histology and serology in these patients (2,13). However, it is not clear to what extend other conditions of the celiac spectrum such as LD respond to a GFD. On the other hand, some studies have shown improvement in patients with dyspepsia or IBS like symptoms and non-specific enteropathies following a GFD suggesting that a GFD might have more implications than just treatment of classic CD patients (14-16). The aim of the present study was to determine the distribution of gluten sensitivity spectrum in a sample of patients seeking medical attention for chronic significant gastrointestinal symptoms from the celiac spectrum and evaluate their symptomatic, histopathologic and serologic response to a GFD. 

## Patients and Methods

This descriptive observational study was carried out on patients with 15 to 45 years of age, who referred to gastroenterology clinic of Modarres hospital from April 2013 to April 2014 for evaluation of chronic significant complaints of low weight, greasy stool, diarrhea and/or excessive bloating. Patients with a positive H. pylori serology and history of autoimmune diseases were excluded. Patient with IBS were not excluded but due to low number of patients fulfilling the rome-3 criteria, they did not enter in data analysis of the study. A total of thirty-eight patients were recruited during the study period of whom, three were excluded due to non-compliance. Informed written consents were taken from all participants and Ethics and Research Committees of the University approved the study protocol.

Demographic characteristics of the patients as well as a thorough medical history were recorded, followed by a complete physical examination. A symptom was assigned to a patient when he/she mentioned its severity as “significant” or “very severe”. All the thirty-five patients underwent upper endoscopy with four samples of duodenal biopsy as well as serology for assessment of anti-tTG while using normal diet i.e. gluten rich diet.

The endoscopic signs reported suggestive of CD included: mosaic pattern of the duodenal mucosa and scalloping of the valvulae conniventes (5). Intraepithelial lymphocytes (IEL) more than 25 per 100 enterocytes were considered abnormal and if associated with villous atrophy and positive serology for anti-tTG was defined as CD. IEL>25 per 100 enterocytes with normal villous architecture and negative serology was defined as LD (6). 

Patients were categorized in three groups according to their baseline serology and pathology results: 1. Celiac disease (positive serology and villous atrophy), 2. Lymphocytic duodenosis (increased intraepithelial lymphocytes with no villous atrophy), 3. NSD (nonspecific duodenitis defined as infiltration of inflammatory cells and not fulfilling the criteria of CD or LD). 

 After obtaining the baseline data, all patients were put on GFD for six months. Afterwards, patients’ assessments of their symptoms and physical examination results as well as histopathologic and serologic responses to GFD were evaluated. Increase of at least 10 percent in BMI was considered a positive clinical response to GFD. Serology and endoscopy with biopsy were repeated in all patients. Features suggestive of improvement included negative serology, resolution of endoscopic signs mainly disappearance of scalloping and completely normal histopathologic study of the biopsies. 

Data were analyzed using SPSS version 16. Results are presented as mean±SD and percentages. Statistical tests used for data analysis included kruskal-wallis and McNemar. A p-value <0.05 was considered statistically significant. 

## Results

A total of 35 patients (15 male, 20 female, mean (±SD) age: 25.49±6.6) were studied. Of the study population, 14.3% (n=5) had celiac disease (CD), 25.7% (n=9) had lymphocytic duodenosis (LD) and the remaining 60% (n=21) had nonspecific duodenitis (NSD) based on histopathologic and serologic studies. Mean age and gender distribution didn’t vary significantly between these 3 groups. Mean BMI of the 35 was 17.26±0.68 with 80% (n=28) of the patients having a lower than normal (<20) BMI. Of the 28 patients with low BMI, 71.4% (n=20) were in NSD group, 17.8% (n=5) in LD group and 10.7% (n=3) in CD group. Mean BMI was not significantly different between the 3 groups. 

In general the most common complaint reported by 62.8% (n=22) of the patients was bloating and excessive gass, followed by diarrhea in 25.7% (n=9) patients. Two patients who were both categorized in CD GROUP only reported greasy stool. Table 1 summarizes patients’ age, baseline BMI, frequency of low BMI and symptoms in each of the three groups of the study while on gluten rich diet. At the beginning of the study, 14.2% (n=5) patients were serology positive (CD patients), and endoscopic signs (scalloping) were reported in 20% (n=7) patients (3 in CD; 2 in LD and 2 in NSD). 

**Table 1:** Age, BMI and symptoms of the patients at the beginning of the study

	NSD N=21(%)	LD N=9(%)	CD N=5(%)	Total N=35(%)
Age	25±6	28±8	23±5	25±7
BMI	17.2±0.4	17.4±0.9	17.1±1.2	17.3±0.7
Low BMI	21(95.2)	5(55.6)	3 (60)	28 (80)
Bloating	10(47.6)	8(88.8)	4 (80)	22 (62.8)
Greasy stool	-	-	2 (40)	2 (40)
diarrhea	5 (23.8)	2(22.2)	2 (40)	9(25.7)

After six months of GFD, a significant increase was found in total mean BMI (17.3±0.7 v.s. 17.9±0.9) (p=0.042). LD groups showed the most increase in BMI (17.4±0.9 v.s. 18.1±0.7), although the difference with other groups was not found statistically significant. The percentage of patients with low BMI decreased from 82.8% (n=29) to 57.1% (n=20) patients after six months of GFD. This reduction in the frequency of low BMI was most considerable in NSD group. Given that an increase more than 10% in BMI is considered a significant clinical response, of the total 21 patients who showed a rise in BMI after six months of GFD, 10 met the criteria for significant clinical response (i.e. > 10% increase in BMI) including 5 patients in NSD group, 3 in LD group and 2 in CD group. Patients’ subjective assessment of their complaint was recorded after the study period. 

**Table 2:** Comparison of study parameters between baseline and after gluten-free diet in each of the diagnostic groups

	NSD (n=21)		LD (n=9)		CD (n=5)	
	Baseline	After GFD	Baseline	After GFD	Baseline	After GFD
BMI	17.2±0.4	17.8±0.9	17.4±0.9	18.1±0.7	17.1±1.2	17.9±0.7
Low BMI (<20)	21 (100%)	14 (66.6%)	5 (55.5%)	4 (44.4%)	3 (60%)	2 (40%)
Bloating	10 (47.6%)	4 (19%)	8 (88.8%)	5 (55.5%)	4 (80%)	1 (20%)
Diarrhea	5 (23.8%)	4 (19%)	2 (22.2%)	1 (11.1%)	2 (40%)	1 (20%)
Greasy stool	-	-	-	-	2 (40%)	-
Anti-tTG	-	-	-	-	5 (100%)	1 (20%)
Endoscopy	2 (9.5%)	0	2 (22.2%)	1 (11.1%)	3 (60%)	1 (20%)
Normal pathology	-	10 (47.6%)	-	4 (44.4%)	-	3 (60%)

A significant decrease was reported in bloating and excessive gas complaint (62.8% vs 28.5%) (p=0.001). Diarrhea was decreased from 25.7% (n=9) to 17.1% (n=6) patients and the 2 cases of greasy stool were both resolved. 

Of the 5 patients with positive anti-tTG at the beginning of the study, only one remained serology positive after six months of GFD. Repeat of endoscopic study showed that following six month of GFD the number of patients with positive features declined from 7 to 2 and pathologic examination of the biopsies showed a complete resolution (completely normal histopathologic view) in 48.5% (n=17) patients, 10 in NSD group, 4 in LD group and 3 in CD group. Table 2 compares the study parameters before and after the study with respect to the diagnostic categories. 

## Discussion

A total of 35 patients with chronic complaints including low weight, bloating, diarrhea and greasy stool were evaluated using serology, endoscopy and histopathology and were accordingly assigned into three diagnostic groups of CD, LD and NSD. Most of the cases were diagnosed with NSD, followed by LD and CD, respectively. Given the small sample of our study, CD patients formed a notably high proportion of the patients (5/35, 14.2%). This becomes even more considerable when including LD patients as well (14/35, 40%), since both CD and LD are lymphocytic enteropathies, which is in consistent with previous studies mentioning the increasing incidence of the disease and the fact that the spectrum of gluten sensitivity is much wider and more prevalent than expected (2, 4, 5, 7, 8, 9, 10). The values found in our pilot study highlight the necessity for a nation-wide study to obtain the prevalence of the gluten-related disorders in the country. 

We found no difference in age and gender of the patients in each of the three groups. The majority of our study population (80%) had low BMI (<20) regardless of their diagnosis and although the mean BMI was lowest in CD patients, the difference with other groups was not statistically significant. The most frequent complaint was bloating in general and in each of the diagnostic groups followed by diarrhea. After serologic and histopathologic studies, the majority of patients with these two presentations were categorized in NSD group followed by LD group. Despite bloating is not considered a classic symptom for CD, it has been reported very common in other studies too, both in CD and other gluten-related conditions (7, 14 ); while greasy stool and diarrhea and weight loss as classic features of malabsorption were presented together in only 2 patients in our study who were both diagnosed with CD. Consistently, Kabbani et al. reported that classic symptoms of malabsorption are highly suggestive of CD while non-specific symptoms (most commonly bloating in our study) as well as negative anti-tTG serology increases the likelihood ration of NSGS as the diagnosis (17). Walker et al. showed that complaints such as weight loss and poor appetite were significantly correlated with positive anti-tTG test and most GI symptoms such as diarrhea; abdominal pain and dyspepsia were negatively associated with increased IELs in deudonal biopsies (6). None of the symptoms in our study were correlated with a particular diagnosis or serology result. 

Abnormal endoscopic view reported by an expert experienced endoscopist in the field, was most common in CD patients (60%) followed by LD patients (22.2%). While only 9.5% of NSD patients showed the classic scalloping sign. Although endoscopic views are not specific for diagnosis of CD or gluten related lymphocytic enteropathies for that matter, they can be suggestive of these conditions (2, 5). 

Excluding gluten from the diet is the only accepted treatment for CD with already proven effects on all symptoms and complications of the disease. Meanwhile, there is no consensus on the impact of GFD on other forms of the gluten sensitivity spectrum. Recent literature review shows that there is a condition known as gluten intolerance but the diagnostic criteria and categorization are not known yet. Non-celiac enteropathies (LD, NSD), which we evaluated in this study might be part of this spectrum. It has been shown that some of the patients with IBS presentations and dyspepsia with or without borderline entheropathies have responded symptomatically to a GFD (14, 18, 19). However, these studies have mostly shown symptomatic improvement as a response to GFD. In our study, we evaluated endoscopic and histopathologic response as well as symptomatic response in patients with LD and NSD. 

Although all symptoms were improved in general, bloating was the only statistically significant one which, is in consistent with previous findings according to Newnham’s review (14). We found that a considerable proportion of patients with LD and NSD showed symptomatic response to a 6-month period of GFD (3 out of 9, 33.3%; and 6 out of 21, 28.5%; respectively). Moreover, a significant number of patients in LD and NSD groups had completely normal histopathology following GFD (10 out of 21, 47.6% and 4 out of 9, 44.4%, respectively). There were several cases with partially resolved pathologic study when compared with baseline. However, due to the lack of an established threshold, we preferred to only report completely normal biopsies. 

This must be taken into account that, as shown by several studies, LD has more common etiologies other than gluten sensitivity including drugs, infections, immunologic disease, etc. (6, 12, 20). Although we didn’t perform a thorough investigation to rule out the other causes of LD as well as NSD etiologies which might be a limitation of our study, still a considerable number responded to a GFD who are probably those with gluten-responsive presentations. If we only consider the subjects in LD and NSD groups who showed a symptomatic and histopathologic response to GFD as patients with “gluten-sensitive enteropathies”, the prevalence of this condition excluding CD in our study is 46.6%. This is much higher than the 19.7% frequency reported by Santolaria et al. (19). They found that 91.9% of the patients with dyspepsia who started a GFD, showed a symptomatic response while 87.5% of them had a histopathologic or serologic response. Of the total 30 cases of LD and NSD in our study that started a GFD, 30% showed a symptomatic response and 46.6% showed a histopathologic response. None of the study parameters were significantly associated with being respondent to GFD in LD and NSD groups; however, all patients in this category had low BMIs. 

A significant increase in BMI was observed in all 3 groups of the study after 6 months of GFD. Interestingly, the highest level of increase was found in LD group. The beneficial significant effect of GFD in BMI level of CD patients was already well documented. However, data on the impact of GFD on other conditions of gluten sensitivity such as LD is less frequent. After correcting for more than 10% increase in BMI as the accepted level for clinical response, still 23.8% of NSD group and 33.3% of LD group showed significant BMI increase showing the extent of the GFD impact. After 6 months of GFD, 4 of the 5 CD patients became seronegative. This is in consistent with several previous studies that have shown disappearance of anti-tTG even following GFD. Histopathological response to GFD was also remarkable in our study. A completely normal pathologic study was found in 10 of the 21 NSD patients, 4 of the 9 LD patients and 3 of the 5 CD patients following a GFD. 

In a nutshell, we found that a high proportion of patients seeking medical attention with non-specific presentations might have gluten-related enteropathies including in the categories of CD, LD and NSD, demonstrating the need for a large scale population-based study to determine a close estimation of these conditions in the country. In addition, a considerable number of patients in LD and NSD groups respond both clinically and histopathologically to exclusion of gluten from diet. Except for low BMI, we couldn’t determine the characteristics of those LD and NSD patients who responded to GFD due to small study population. It seems that patients with NSD and LD who have low BMI might benefit the most from a GFD. Moreover, our study lacked HLA typing as a very accurate test for diagnosis of genetically susceptible cases to gluten since this test is expensive and not readily available in our country. Further studies are needed to confirm our findings so that the physicians can make an evidence-based decision on prescribing a GFD for patients with these conditions.
